# Grappling with grapevine guardians: Unraveling the dynamic dance of VviWRKY10 and VviWRKY30 in powdery mildew defense

**DOI:** 10.1093/plphys/kiae095

**Published:** 2024-02-22

**Authors:** Ritu Singh

**Affiliations:** Assistant Features Editor, Plant Physiology, American Society of Plant Biologists; Department of Plant Science, University of California, Davis, CA, 95616, USA

Plants face constant threats from various microbial pathogens, posing a significant risk to global food security. To counter these threats, plants have evolved sophisticated defense mechanisms primarily involving physical and chemical strategies to protect themselves against these microbes (Jones and Dangl 2006; Ramirez-Prado et al. 2018). In addition to these preformed physical/chemical barriers, plants actively detect pathogens and trigger defense signaling networks, restricting the further growth and spread of pathogens. Transcription factors (TFs) play a central role in these signaling networks by governing the expression of defense genes. Exploring TF regulation during plant-pathogenic fungi interactions opens avenues to discover novel virulence factors and enhance our understanding of the regulatory networks associated with pathogen evolution. Moreover, targeting TFs themselves can be a strategy for disease control.

Grapevine (*Vitis vinifera*) is one of the most economically important fruits globally. However, its production is severely hampered by *Erysiphe necator*, an obligate biotrophic fungal pathogen that causes powdery mildew disease. Although previous research identified *AtWRKY18* and *AtWRKY40* as negative regulators of powdery mildew resistance in *Arabidopsis* (Xu et al. 2006, Pandey et al. 2010), the specific role of WRKY TFs in grapevine remained elusive.

In the recent *Plant Physiology* issue, Zhou and colleagues (2024) explored the function of *VviWRKY10* and *VviWRKY30* (orthologs of *AtWRKY18* and *AtWRKY40*) in grapevine powdery mildew resistance. The authors used the CRISPR-Cas9 method to generate single and double mutant lines in the powdery mildew–susceptible Cabernet Sauvignon grape cultivar. They successfully obtained 53 *wrky10* mutants and 15 *wrky10wrky30* mutants, with no *wrky30* mutant plants. Upon infection with *E. necator* NAFU1, *wrky10* plants displayed strong resistance, whereas *wrky10wrky30* showed moderate resistance compared to wild-type plants. Further, trypan blue, 3,3'-diaminobenzidine, and aniline blue staining revealed enhanced hypersensitive response–like cell death, H_2_O_2_, and callose accumulation, respectively, in *wrky10* lines, moderate in *wrky10wrky30*, and none in wild-type.

Because no *wrky30* CRISPR-Cas9 mutants were obtained, the authors transiently overexpressed VviWRKY30 along with VviWRKY10 in Cabernet Sauvignon leaves through Agrobacterium-mediated transformation. Leaves overexpressed with VviWRKY30 showed increased resistance against powdery mildew, whereas leaves overexpressed with VviWRKY10 exhibited more susceptibility compared to the wild type, indicating their opposite roles in powdery mildew resistance. Further, real-time expression analysis identified that *VviWRKY10* was induced at the early stage of infection (peaking at 24 hpi), whereas *VviWRKY30* was induced at a later time (peaking at 48 hpi), suggesting their distinct roles in the transcriptional regulation of host defense at different infection stages.

WRKY TFs can modulate the levels of salicylic acid (SA) and ethylene (ET) hormones during plant–pathogen interactions (Dang et al. 2014; Birkenbihl et al. 2017). Zhou et al. (2024) selected key genes involved in SA and ET hormone signaling and response and measured their expressions in *E. necator*-inoculated mutants and wild-type plant leaves. The result indicated significantly increased expression of most of these genes in both *wrky10wrky30* and *wrky10*, with a more specific upregulation in *wrky10* lines. To further validate this, the authors quantified SA and ET levels in leaf tissues of wild-type and mutant lines, which confirmed higher SA levels in both mutants and elevated ET in *wrky10*. This suggests that VviWRKY10 and VviWRKY30 influence grapevine powdery mildew resistance by altering SA and ET.

WRKY TFs specifically bind to W-box (TTGACC/T) elements on the promoters of the target genes (Rushton et al. 2010). Chromatin immunoprecipitation-quantitative real-time PCR assays and electrophoretic mobility shift assays analysis demonstrated that VviWRKY10 and VviWRKY30 could directly bind to the promoters of key genes in SA and ET pathways, such as *VviEDS5-2*, *VviPR1*, *VviPR5*, *VviACS3/ACS3L*, and *VviRBOHD2*. Further, dual-luciferase assays suggest that VviWRKY10 inhibited the transcription of SA- and ET-related genes, whereas VviWRKY30 significantly enhanced the transcription of ET-related genes. Moreover, VviWRKY10 and VviWRKY30 can mutually inhibit each other by binding to each other's promoters.

In summary, the study highlights the distinctive roles of VviWRKY10 and VviWRKY30 in powdery mildew resistance by modulating SA and ET hormones (Figure). VviWRKY10 suppresses the SA-dependent defense responses upon powdery mildew infection, whereas VviWRKY30 promotes ET production for limiting powdery mildew growth at a later stage of nfection. Furthermore, VviWRKY10 and VviWRKY30 can inhibit each other to inhibit ET synthesis and SA and ROS production, respectively. The mutual inhibition of VviWRKY10 and VviWRKY30 and their distinct functions might be crucial for balanced defenses through regulating multiple hormonal pathways against powdery mildew and potentially other pathogens in grapevines. Additionally, these genes appear to be linked to plant growth, as mutants displayed reduced height and growth compared to the wild type, highlighting the intricate relationship between growth and defense. These genes stand out as valuable tools for studying this delicate balance.

**Figure.**
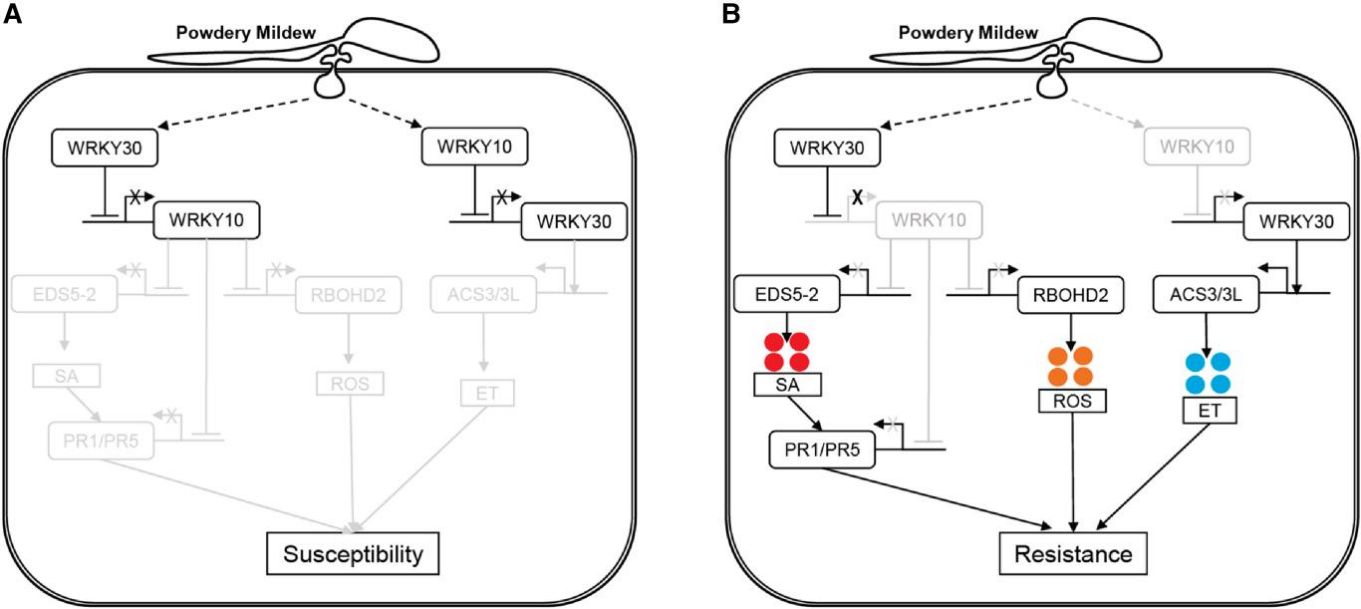
A proposed working model of VviWRKY10 and VviWRKY30 (Fig. 9 A, B of Zhou et al. 2024). **A)** VviWRKY10 and VviWRKY30 interact by binding to each other's promoters, leading to mutual inhibition. VviWRKY10 suppresses VviWRKY30, thereby inhibiting ethylene synthesis, while VviWRKY30 suppresses VviWRKY10, hampering salicylic acid and reactive oxygen species production. Consequently, this regulatory interaction renders plants vulnerable to powdery mildew disease. **B)** In instances where WRKY10 expression/activity is absent, WRKY30 remains uninhibited, promoting ethylene production. Additionally, the lack of active WRKY10 results in increased salicylic acid and reactive oxygen species levels. This results in enhanced resistance against powdery mildew in grapevine.

## Data Availability

No new data were generated or analysed in support of this research.

## Dive Curated Terms

The following phenotypic, genotypic, and functional terms are of significance to the work described in this paper:

WRKY10 Gramene: PP236376
WRKY10 Araport: PP236376

WRKY30 Gramene: PP236377
WRKY30 Araport: PP236377
